# Epigenetic manipulation for secondary metabolite activation in endophytic fungi: current progress and future directions

**DOI:** 10.1080/21501203.2023.2241486

**Published:** 2023-08-08

**Authors:** Ashish Verma, Harshita Tiwari, Swati Singh, Priyamvada Gupta, Nilesh Rai, Santosh Kumar Singh, Bhim Pratap Singh, Sombir Rao, Vibhav Gautam

**Affiliations:** a Centre of Experimental Medicine and Surgery, Institute of Medical Sciences, Banaras Hindu University, Varanasi, India; b Department of Botany, Institute of Science, Banaras Hindu University, Varanasi, India; c Department of Agriculture & Environmental Sciences (AES), National Institute of Food Technology Entrepreneurship & Management (NIFTEM), Sonepat, India; d Plant Breeding and Genetics Section, School of Integrative Plant Science, Cornell University, Ithaca, NY, USA

**Keywords:** Fungal endophytes, chromatin, histone modification, bioactive compounds, secondary metabolites

## Abstract

Fungal endophytes have emerged as a promising source of secondary metabolites with significant potential for various applications in the field of biomedicine. The biosynthetic gene clusters of endophytic fungi are responsible for encoding several enzymes and transcriptional factors that are involved in the biosynthesis of secondary metabolites. The investigation of fungal metabolic potential at genetic level faces certain challenges, including the synthesis of appropriate amounts of chemicals, and loss of the ability of fungal endophytes to produce secondary metabolites in an artificial culture medium. Therefore, there is a need to delve deeper into the field of fungal genomics and transcriptomics to explore the potential of fungal endophytes in generating secondary metabolites governed by biosynthetic gene clusters. The silent biosynthetic gene clusters can be activated by modulating the chromatin structure using chemical compounds. Epigenetic modification plays a significant role by inducing cryptic gene responsible for the production of secondary metabolites using DNA methyl transferase and histone deacetylase. CRISPR-Cas9-based genome editing emerges an effective tool to enhance the production of desired metabolites by modulating gene expression. This review primarily focuses on the significance of epigenetic elicitors and their capacity to boost the production of secondary metabolites from endophytes. This article holds the potential to rejuvenate the drug discovery pipeline by introducing new chemical compounds.

## 1. Introduction

Endophytic fungi are well-known for their interaction with a diverse group of medicinal plants resulting in the production of secondary metabolites (SMs). Studies have evidenced that fungal endophytes serve as a prolific producer of a wide variety of pharmaceutically important SMs that exhibit immense therapeutic potential (Raimi and Adeleke 2021; Baron and Rigobelo 2022). The symbiotic relationship between plant and fungal endophytes has attracted enormous scientific attention due to their versatile nature and intriguing biomedical significance (Singh et al. 2021; Rai et al. 2022). The focus of scientific community towards endophytic microbiota was shifted after the discovery of paclitaxel, a derivative of taxol, from a fungal endophyte *Taxomyces andreanae*, inhabiting the innermost layer of bark of *Taxus brevifolia* (Pacific Yew tree) (Stierle et al. 1993). Taxol is a well-known anti-cancer drug derived from the bark of *Taxus brevifolia*, which is widely used for the treatment of various types of cancer including lung, breast, and ovarian cancer (Ahn et al. 2004). The potential of fungal endophytes and their establishment as therapeutic sources were sparked by the fact that they mimicked host metabolites, as explained by many theories such as balanced-antagonism theory and horizontal-gene transfer (Tiwari and Bae 2022; Srinivasa et al. 2022). The bioactive potential of some fungal endophytes has since been the subject of numerous studies (Strobel 2002; Zhang et al. 2006; Suryanarayanan et al. 2009), in an effort to gain a better understanding of it. Fungal endophyte produces SMs of various classes, such as phenolics, flavonoids, steroids, terpenoids, polyketides, quinones, and xanthones, which are being used in the pharmaceutical, cosmetic, industrial, and agrochemical fields (Mousa and Raizada 2013; Uzma et al. 2018; Gupta et al. 2020). The derived SMs exhibit antagonistic effect against certain plant pathogens and protect their host plant by eliciting defence mechanism and supporting their growth and survival. Many of these metabolites have been subjected to biochemical characterisation and assays for their recognition as potent source of novel compounds of various medical significance. In one of the previous reports, a fungal endophyte *Diaporthe longicolla* isolated from the leaf of *Saraca asoca*, has been shown to produce several bioactive compounds with antibacterial and antioxidant properties (Nishad et al. 2021). A recent study has also revealed that ethyl acetate extract of endophytic fungus *Nigrospora sphaerica* possess significant antioxidant activity against synthetically generated free radicals (Gautam et al. 2022). An endophytic fungus, *Phyllosticta capitalensis* has also been studied for the production of numerous bioactive compounds with antibacterial and antitumour activities (Xu et al. 2021). In one of the reports, *Rosellinia* sp., a fungal endophyte residing in a Japanese tea plant, has been shown to produce a compound, PF-1022A, a nematocidal cyclodepsipeptide which was used for the semisynthetic synthesis of antiparasitic drug, emodepside (Helaly et al. 2018). Despite having exceptional metabolic capacity, SMs gene clusters or biosynthetic gene clusters (BCGs) of fungal endophytes express partially in the *in vitro* conditions (Toghueo et al. 2020). The exploration of fungal endophytes for obtaining targeted metabolites or novel drug discovery is therefore a difficult and tedious process due to certain limitations. Biogenetic capacity of microorganisms can be enhanced by the application of epigenetic modifications and other genomic analysis (Brakhage and Schroeckh 2011; Marmann et al. 2014). The term “epigenetic” describes heritable modifications in gene expression that take place without altering the DNA sequence. The putative BGCs are located in the heterochromatin region of chromosome and therefore remain silent. However, the activation and deactivation of these regions are regulated by molecular mechanisms including histone acetylation and DNA methylation. Epigenetic regulation or modification through alteration of acetylation, methylation and induced changes in histones leads to the activation of cryptic gene clusters and production of novel chemical entities (Akone et al. 2019). Treatment of fungal endophytes by epigenetic elicitors has been validated as a better approach for the activation of biosynthetic gene clusters (BGCs) that are responsible for the production of SMs. Interestingly, chemical elicitors have been discovered to activate these silent cryptic genes to trigger the production of SMs of fungal endophytes. Epigenetic modulation can be achieved by the application of DNA methyltransferase inhibitors [hydralazine hydrochloride, 5-azacytidine (**1**) (5-AZA), and 5-aza-2’-deoxycytidine (**2**)] and several histone deacetylase inhibitors [suberohydroxamic acid (**3**) (SBHA), sodium butyrate (**4**), suberoylanilide hydroxamic acid (**5**) (SAHA), trichostatin A (**6**), and valproic acid (**7**)] (Xue et al. 2023).

Several omics-based approaches such as genomics, proteomics, transcriptomics, and metabolomics can serve as potential strategies to comprehend the molecular and biochemical applications of BGCs (Barik et al. 2020; Keshri et al. 2021; Salvi et al. 2022). Epigenetics, which is formerly used for gene regulation, can be further exploited as a biotechnological tool due to which it would be a better approach to use epigenetics for understanding biogenetic capabilities of fungal endophytes to harvest SMs. The current review article emphasises the activation of biosynthetic gene clusters via epigenetic modulations by the application of epigenetic elicitors. Furthermore, this article sheds light on the cutting-edge genome editing method CRISPR-Cas9, which shows immense promise as a revolutionary approach in fungal endophytes research for modifying their metabolite profiling.

## 2. Exploring the biosynthetic gene clusters of fungi for secondary metabolite production

BGCs are group of genes involved in the production of SMs. Discovery and availability of various omics-based approaches have revolutionised and fastened the BGCs discovery pipeline. The expression of BGCs that encode for SMs production is generally regulated by environmental factors and based on the growth phase of the fungus (Keller 2019). The fungal genomics also support the scientific hypothesis that BGCs are responsible for the production of promising molecules and various chemicals (Rokas et al. 2020). Advancements in sequencing technologies have provided a novel platform to access fungal genomes, thereby offering insights into the biosynthetic capabilities of fungi (Osbourn 2010). These gene clusters typically include genes for attenuating enzymes that alter the core structure of the backbone enzymes of the compound, regulatory proteins, such as transcription factors and gene encoding resistance (Brakhage 2013). Furthermore, advancements in genomics have challenged the validity of this theory regarding the biosynthetic pathways of SMs produced by fungal species and have established two fundamental concepts. The first concept revolves around BGCs, which consist of genes grouped together on a single genetic locus, and they encode the enzymes necessary for the production and transportation of SMs (Keller et al. 2005; Khaldi et al. 2010). The design of the genes responsible for producing fungal SMs has made it possible to create computer algorithms that can forecast the primary enzymes required for producing different classes of SMs (Keller 2019). On the other hand, the latter concept proposes that for any fungal strain, there are significantly more BGCs than there are SMs (Hoffmeister and Keller 2007; Rutledge and Challis 2015). The main reason for the discrepancy between the actual number of BGCs and the number of recognised chemical entities synthesised by any particular fungus is that most BGCs are transcriptionally inactivated or moderately expressed in proper laboratory conditions (Hoffmeister and Keller 2007; Schüller et al. 2020). Hence, to improve SMs production in fungal endophytes, it is necessary to develop methods that may be employed to trigger the stimulation of silent or cryptic biosynthetic pathways.

## 3. Unravelling biosynthetic gene clusters: techniques for identification and characterisation

An effective method to determine the active BCGs is expression-based analysis (Schley et al. 2006; Udwary et al. 2011). The two different proteomics-based studies have been evolved for the identification of proteins encoded by BGCs, either by affinity purification methods (Meier et al. 2009) or by the identification of the target of reporter ions, non-ribosomal peptide synthases (NRPS) and polyketide synthases (PKS) using mass spectrometry (Bumpus et al. 2009), to modify amino acids with the essential phosphopantetheine. In case of non-availability of genome, the sequencing of peptides can be done from the scratch for the synthesis of degenerate primers to amplify BGCs via polymerase-chain reaction (PCR). The PCR method can also amplify cDNA using degenerate primers encoding sequences frequently present in BGCs (Qu et al. 2011). The BGCs that enable strain selection to induce epigenetic modifications have been analysed and predicted using an extensive genome library. Using *in-silico* modelling of potential physicochemical characteristics of their end products, the available bioinformatics packages have not merely provided the estimation of the assembly line but additionally the substrates. Various tools have been used for strain prioritisation and exploration including antibiotics & Secondary Metabolite Analysis Shell (anti-SMASH) (Weber et al. 2015), PRediction Informatics for Secondary Metabolomes (PRISM) (Jiang et al. 2012), environmental Surveyor of Natural Product Diversity (eSNaPD) (Reddy et al. 2014), and ClusterMine360 (Conway and Boddy 2012). The chromatin immunoprecipitation sequencing (ChIP-seq) (Park 2009), ChIP-chip (Zhang et al. 2008), MeDIP-seq (Taiwo et al. 2012), and Whole-Genome Bisulfite Sequencing (Xi and Li 2009) are a few of the software programs designed to evaluate epigenomics data relevant to investigating the levels of methylation and differentially methylated areas.

## 4. Methods for probing secondary metabolism and chromatin modification in fungi

**Figure 1.**
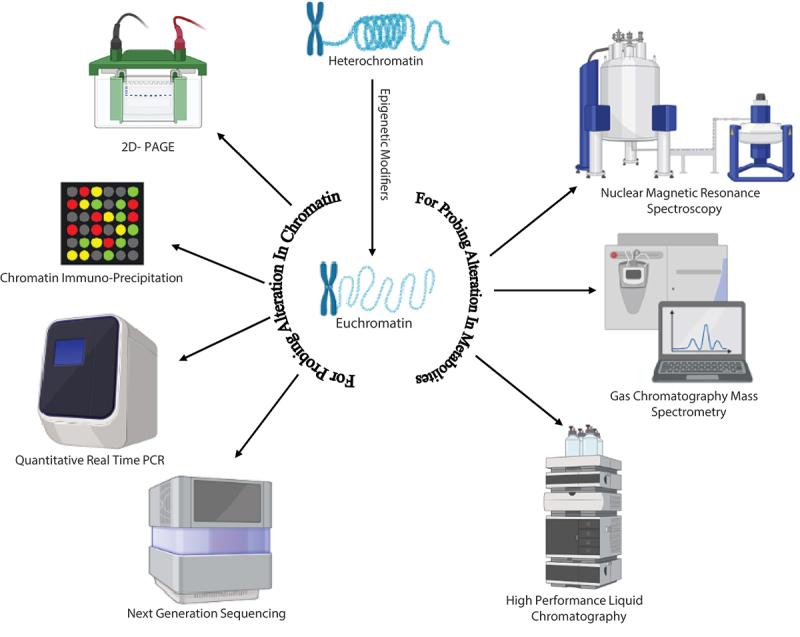
The schematic representation showcases various approaches utilised in the study of chromatin modification. Techniques such as HPLC (High-Performance Liquid Chromatography), GC-MS (Gas Chromatography-Mass Spectrometry), and NMR (Nuclear Magnetic Resonance) spectroscopy are employed to gather information regarding alterations in metabolite profiling. These techniques contribute valuable insights into the characterisation and analysis of chromatin modifications.

Most research on chromatin alterations and their relevance to secondary metabolism relies on two key approaches. The first approach involves measuring the overall levels of histone alterations, generally by western blotting. Highly efficient western blotting procedures will benefit histones by the aid of nucleus isolation, purification (Soukup and Keller 2013) or acid extraction (Jourquin and Géli 2017). The isolated histones are subjected to poly acrylamide gel electrophoresis (PAGE) and analysed using antibodies specific to a particular histone alteration. Antibodies to the C-terminus of histone octamer subunit H3 or H4 that have not been changed are frequently utilised as loading controls. Mass-spectrometry technique helps to identify the SMs of fungal endophyte and also imply to measure the concentrations of the post-translational modifications in histones because of advancements in mass spectrometry (MS) methodologies and procedures (Krautkramer et al. 2015; Gupta et al. 2021). Although, it is difficult to quantify specific alterations using MS because the applicability of this technique for filamentous fungus is yet to be explored. However, variation in the methodology showed that the MS could pick up alterations in a small number of histones PTMs in *Aspergillus nidulans* (Gacek-Matthews et al. 2015, 2016). The histone PTMs at particular loci could not be revealed by western blotting or MS, even though they can offer information on changes in global histone modification levels. Instead, ChIP method is used to detect the extent of modifications in histone protein at specific target and the level of binding of crucial proteins to DNA either directly or indirectly (Orlando 2000). Different techniques for identifying alteration in chromatin modification and upregulation of SMs or novel compounds production have been represented in Figure 1. When genomic loci are very small in numbers, the quantification of DNA can be done using quantitative polymerase chain reaction (qPCR), or it can be studied on a large scale using advanced techniques such as chip-sequencing technology or microarray technology (ChIP on chip) (Boedi et al. 2012). In addition to RNA-sequencing, histone modification variations across the genome can be examined, and changes can be correlated with transcriptomics. In line with the advancement of ChIP technology, analytical chemistry methods have also changed, such as shifting the microarrays technique towards next-generation sequencing method. A conventional chromatographic method, thin-layer chromatography (TLC) was used to detect variations in SMs biosynthesis in the innovative study that first described the connection between histone PTM and secondary metabolism (Shwab et al. 2007). TLC is no longer employed since high performance liquid chromatography (HPLC) in combination with a range of detectors provides additional knowledge about the SMs of differently mutated or modified chromatin. The most comprehensive examination of the entire metabolome is provided by the utilisation of MS detectors coupled with HPLC or methods like two-dimensional nuclear magnetic resonance (2D-NMR) spectroscopy, which also significantly helps in the isolation of novel molecules (Forseth et al. 2011; Pfannenstiel et al. 2018).

## 5. Epigenetic remodelling of silent gene clusters

Histones are fundamental proteins that help in the packaging of DNA to form nucleosomes and therefore have an epigenetic state directly related to the chromatin structure (Ehrenhofer‐Murray 2004). Among the epigenetic modification by post-translational modification, histones are methylated, acetylated, or phosphorylated serine, ubiquitinated lysine, and methylated arginine. The whole expression of genes is significantly influenced by DNA methylation and chromatin structure modulation, which have a remarkable impact on the biosynthesis of SMs in terms of quality or quantity. This allows the possibility of utilising drugs with epigenetic modification potential to activate silenced cryptic genes’ transcriptional pathways (Williams et al. 2008; Yang et al. 2013). According to various studies, the heterochromatin stage where genes are transcriptionally controlled by reversible epigenetic alterations like acetylation or methylation is the site of the fungal metabolic gene clusters that are typically located in the distal regions of chromosomes (Nierman et al. 2005; Shwab et al. 2007; Fisch et al. 2009). The activation of silent BGCs responsible for the production of SMs has demonstrated the effectiveness of specific epigenetic modulators such as DNA methyltransferases (DNMT), 5-azacytidine (**1**), and histone deacetylase (HDAC). The synthesis of fungal metabolites can be adversely affected by the acetylation and methylation events that can modify histones (Shwab et al. 2007; Bok et al. 2009). Many novel SMs have been discovered during the last 20 years that can subsequently retard fungal growth by the application of HDAC and DNMT chemical inhibitors. Another study found that the supplementation of 5-AZA (**1**) to the nutrient medium of fungal endophyte *Pestalotiopsis crassiuscula*, significantly changed the property of its SMs, leading to the synthesis of a new coumarin known as 4,6-dihydroxy-7-hydroxymethyl-3-methoxymethylcoumarin (Yang et al. 2014). Researchers also observed a comparable ‘chemical change’ when they used 1 μmol/L of 5-azacytidine in culture medium to cultivate an endophytic fungus *Aspergillus niger* (Toghueo et al. 2016). Another study found that hydralazine hydrochloride can activate metabolic pathways that produce different SMs by decreasing the DNA-methylation-mediated suppression of several genes and cellular functions in different fungal strains (Cichewicz 2010). When fungi are treated with epigenetic modifiers, the cultural characteristics of a particular strain are also influenced in addition to the metabolite profile. In fact, *Hypoxylon* sp. CI-4 showed striking shifts in its culture parameters, which involved alterations in pigmentation, efficiency, and odour, including major differences in the biological functioning of the volatile organic compounds (VOCs), when exposed to epigenetic elicitors 5-AZA (**1**) and SAHA (**5**) (Ul-Hassan et al. 2012).

### 5.1. Histone modification: DNA methylation and acetylation in epigenetic regulation

Co-expressed genes, which are frequently localised in the biosynthetic gene clusters, significantly control the synthesis of SMs by fungal endophytes. Because of strict transcriptional regulation, most pharmacological SMs encoding gene clusters are expected to be cryptic in axenic cultures. DNA accessibility is regulated by chromatin modifications, an essential factor in transcriptional regulation. The chromatin complex consists of DNA and the critical histone octamers containing two copies of histone proteins H2A, H2B, H3, and H4, which are conserved in fungal genome regions (Armeev et al. 2019). Chromatins are found in two forms: the heterochromatin stage, when it is densely packed and transcriptionally inactive, or the euchromatin stage when it is loosely packed and transcriptionally active. Additionally, chromatin can respond to various environmental signals. Post-translational epigenetic modifications, such as histone methylation and acetylation, play a role in altering chromatin packaging, thereby influencing the accessibility of DNA to the transcriptional machinery, and ultimately regulating gene expression (Strahl and Allis 2000). DNA methyltransferases promotes covalent addition of methyl group (-CH_3_) on arginine or lysine residue of adenine or cytosine bases, which regulates the translational factors and ribosomal proteins involved in inhibiting gene transcriptional machinery (Iyer et al. 2016; Kumar et al. 2018). Histone acetyltransferases (HATs) facilitate the acetylation of conserved lysine residue of histone octamer, involving the transfer of an acetyl group from acetyl-coenzyme A (Sterner and Berger 2000). An amino acid, lysine, with a negative charge, affects the transcription potential and stimulates transcription by interacting with histone octamers and DNA (Collemare and Seidl 2019). On the other hand, deacetylation by HDACs reduces the amount of readily available DNA by providing positive charge on lysine residue, allowing opposite charge interaction between histone tail and DNA. As a result, the hypoacetylated state of chromatin leads the chromatin to become more condensed (Seto and Yoshida 2014).

**Figure 2.**
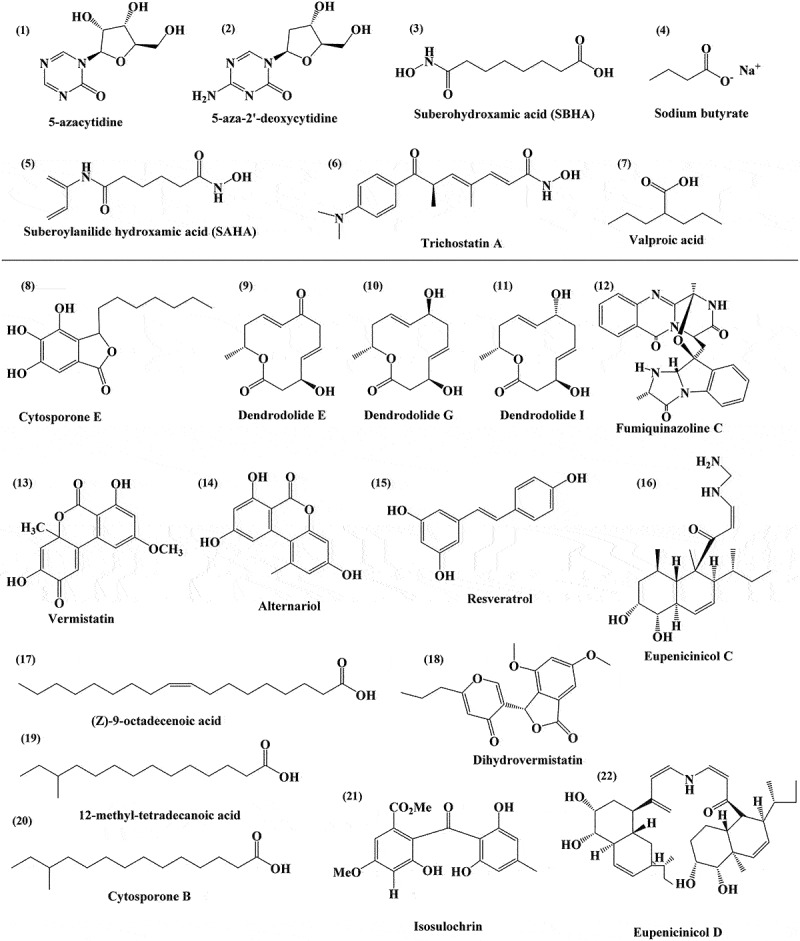
Chemical structure of epigenetic modifiers, such as 5-azacytidine **(1)**, 5-aza-2’-deoxycytidine **(2)**, suberohydroxamic acid (SBHA) **(3)**, Sodium butyrate **(4)**, Suberoylanilide hydroxamic acid (SAHA) **(5)**, Trichostatin A **(6)**, and Valproic acid **(7)** that are being used for the enhancement of secondary metabolite production. The chemical structure of metabolites obtained through epigenetic modifications, such as Cytosporone E **(8)**, Dendrodolide E **(9)**, Dendrodolide G **(10)**, Dendrodolide I **(11)**, Fumiquinazoline C **(12)**, Vermistatin **(13)**, Alternariol **(14)**, Resveratrol **(15)**, Eupenicinicol C **(16)**, (Z)-9-octadecenoic acid **(17)**, Dihydrovermistatin **(18)**, 12-methyl-tetradecanoic acid **(19)**, Cytosporone B **(20)**, Isosulochrin **(21)**, and Eupenicinicol D **(22)**.

Fungal endophytes may significantly alter the biomolecules they make when their DNA is acetylated and methylated by various epigenetic modifiers in a laboratory environment (Shwab et al. 2007; Bok et al. 2009). The grass fungal endophyte *Epichloe festucae* was found to have H3K9 and H3K27 trimethylations, which were claimed to impact the expression of genes regulating the production of bioprotective alkaloid compounds. The development of symbiotic associations among *E. festucae* with perennial ryegrass (*Lolium perenne*) depends on these metabolites (Chujo and Scott 2014). Chujo and Scott, showed silencing of alkaloid biosynthesis genes in *E. festucae* due to epigenetic modifications. They were revealed by the role of H3K9 methylation regulation of alkaloid biosynthesis in fungal endophytes. On the other hand, *in vitro*, suppression of *ltm* and expression of *eas* gene was induced by the silencing of the methyl transferase-encoding genes H3K9-(ClrD) or H3K27-(EzhB) (Chujo and Scott 2014). The names and target sites of the chemical epigenetic modulators for producing specialised SMs by fungi and elicited metabolites are listed in Table 1, and their structures are depicted in Figure 2. In another study, it was observed that the fungal endophyte *Pestalotiaopsis fici* synthesised various unique polyketides including pestaloficiols T – W and 11 macrodiolides (ficiolides A to K) after the deletion of two putatively associated genes for epigenetic regulation such as histone methyltransferase and histone deacetylase (Wu et al. 2016). Another study revealed that the suppression of epigenetic repressor such as histone H3 deacetylase causing the upregulation of BGCs in *Calcarisporium arbuscula* resulting in the overproduction of some well-known bioactive compounds including four novel cyclic peptides such as diterpenoid arbuscullic acid A, arbumelin, arbumycin, and meroterpenoid arbuscullic acid B by the fungal strain (Mao et al. 2015). This research indicates that altering the chromatin structure may be a highly effective method for triggering silent gene clusters of fungal endophytes to produce novel biologically active SMs.

**Table 1.** List of epigenetic modifiers, their mode of action and elicited compounds.

S. No.	Endophytic fungi	Host plant	Epigenetic modifier	Mechanism of action	Elicit compounds	Reference
1.	*Leucostoma persoonii*	*Rhizophora mangle*	5-azacytidine **(1)**	Inhibition of DNA methyl transferase	Cytosporone B **(20)**	Beau et al. (2012)
2.	*Leucostoma persoonii*	*Rhizophora mangle*	Sodium butyrate **(4)**	Inhibition of HDAC of classes I and II	Cytosporone E **(8)**	Beau et al. (2012)
3.	*Dimorphosporicola tragani*	*Arthrocnemum macrostachyum*	5-azacytidine **(1)**, Valproic acid (**7**)	Inhibition of DNA methyl transferase, Inhibition of HDAC of classes I and II	Dendrodolide E **(9)**, G **(10)**, I **(11)**	González-Menéndez et al. (2019)
4.	*Pestalotiopsis crassiuscula*	*Fragaria chiloensis*	5-azacytidine **(1)**	Inhibition of DNA methyl transferase	4,6-dihydroxy-3,7-dimethylcoumarin, pestalotiopyrone G and 2′-hydroxy-6′-hydroxymethyl-4′-methylphenyl 2,6-dihydroxy-3-(2-isopentenyl)benzoate	Yang et al. (2014)
5.	*Aspergillus fumigatus*	*Grewia asiatica*	Valproic acid **(7)**	Inhibition of HDAC of classes I and II	Fumiquinazoline C **(12)**	Magotra et al. (2017)
6.	*Chaetomium* sp.	*Sapium ellipticum*	SAHA **(5)** or 5-azacytidine **(1)**	Inhibition of HDAC of classes I and II, Inhibition of DNA methyl transferase	Isosulochrin **(21)**	Akone et al. (2016)
7.	*Eupenicillium* sp.	*Xanthium sibiricum*	NAD+-dependent histone deacetylase inhibitor	Inhibition of HDAC of class I	Eupenicinicol C **(16)** and D **(22)**	Li et al. (2017)
8.	*Alternaria* sp.	*Datura stramonium*	SAHA **(5)**	Inhibition of HDAC of classes I and II	Alternariol **(14)**, alternariol-5-O-methyl ether, 3′-hydroxy-5-methoxyalternariol, altenusin, (5S, 8S)-tenuazonic acid	Sun et al. (2012)
9.	*Phoma* sp.	*Parkinsonia microphylla*		Inhibition of HDAC of classes I and II	Vermistatin **(13)** and dihydrovermistatin **(18)**	Gubiani et al. (2017)
10.	*Penicillium herquei*	*Cordyceps sinensis*	5-aza-2-deoxycytidine **(2)**	Inhibition of DNA methyltransferase	(S)-6-(sec-butyl)-5-(hydroxymethyl)-4-methoxy-2 H-pyran-2-one and (5S,7 R)-7-ethyl-4,5-dimethoxy-7-methyl-5,7-dihydro-2 H-furo[3,4-b]pyran-2-one	Guo et al. (2020)
11.	*A. niger*	*Terminalia catappa*	5-azacytidine **(1)**	Inhibition of DNA methyl transferase	(Z)-9-octadecenoic acid **(17)** and 12-methyl-tetradecanoic acid **(19)**	Toghueo et al. (2016)

### 5.2. CRISPR-Cas9 genome editing for augmenting secondary metabolite production in fungal systems

**Figure 3.**
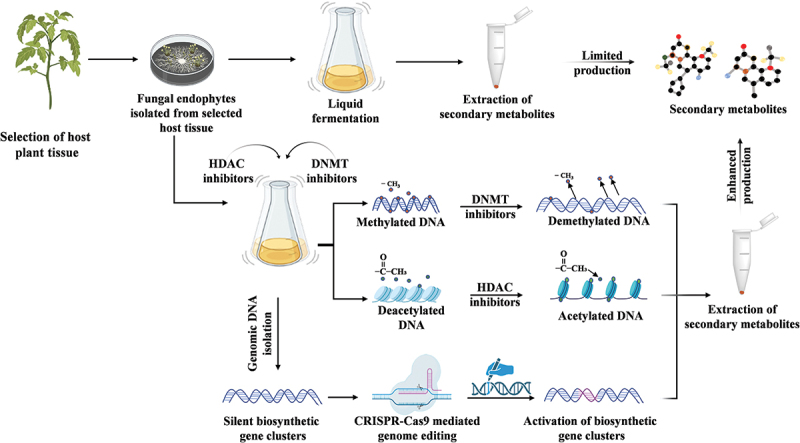
The diagram illustrates different strategies employed to enhance the production of fungal endophyte-associated metabolites. One approach involves the use of chemical elicitors to boost the production of secondary metabolites. Another approach utilises the CRISPR-Cas9 mediated genome editing technique, which enables targeted modifications to the fungal strain’s genome, ultimately leading to enhanced metabolite production.

Modifications at the genomic level comprise alteration in the genome sequence or exogenous DNA insertion to activate cryptic genes, location-specific transgene integration, or removing mutant alleles (Brakebusch 2021). Another cutting-edge technology, genome editing, can alter DNA (by insertion or deletion) and expand the possibility of heavy metal mycoremediation. Guide sequences are created during the complementary process to the target sequence and help to identify the breaking spot and facilitate homologous recombination repair. Earlier studies indicated that the three most widely employed methods for genetic engineering, utilized to modify the genomes of living organisms, include (i) Clustered Regularly Interspaced Short Palindromic Repeats and CRISPR-associated protein (CRISPR-Cas9), (ii) Transcription Activator-Like Effector Nucleases (TALEN), and (iii) Zinc-finger nucleases (ZFN). These techniques are currently utilized as genetic engineering tools to modify the genomes (Rai et al. 2021). The technology used for the modifications that stands out the most is CRISPR-Cas technique. The recent development in the cutting-edge gene-editing tools, particularly the CRISPR/Cas system, has transformed high-throughput epigenetic modification in fungal endophytes by overcoming the hurdles and creating novel pathways for producing significant SMs (Figure 3). CRISPR-based method was first identified as an antiviral immune defence mechanism in most bacterial species (Barrangou et al. 2007; Jinek et al. 2012). CRISPR-Cas systems are classified into two classes, that is class I and class II, in which each class includes six different varieties, as per the most recent evolutionary classification criteria (Makarova et al. 2020). The Class 2 CRISPR/Cas technique has been deeply studied and utilised for genome editing. It comprises the CRISPR-derived RNA (crRNA), Cas9 endonuclease, and the trans-activating CRISPR RNA (Swartjes et al. 2020). A double-strand nick is created on the target sequence of DNA when Cas9 makes a cut in the double-stranded DNA under the guidance of a hybrid crRNA-tracer RNA (Gasiunas et al. 2012). The SM pathway has been successfully modified in fungi by several CRISPR-based techniques, including genome editing, transcriptional control, and epigenetic alteration.

Since its invention, CRISPR-Cas9 has redefined the editing of the genome (Sarma et al. 2021). It has improved the accuracy of modifying genetic sequences in the scientific community (Knott and Doudna 2018). The single guide RNA (sgRNA) and the Cas9 endonuclease are the two key components of the CRISPR-Cas9 type II system for targeting genes and their cleavage. As a result, CRISPR-Cas9 system consisting of chimeric guide RNA and Cas9 endonuclease enables the initiation of nicks in double-stranded DNA, which may then be used to activate gene targeting effectively. The protospacer sequence of the sgRNA allows precise localisation of the sgRNA Cas9 endonuclease to the target site of DNA. The system of cell repair enzymes is initiated when Cas9 endonuclease binds to the target and makes a double-stranded nick. This non-homologous end joining (NHEJ) recombinational repair machinery ensures that the nicks are perfectly sealed, preventing additional insertions or deletions of nucleotides thereby resulting in the arrest of the transcription process of the gene of interest (Bono et al. 2015).

Consequently, CRISPR-Cas9-based editing facilitates a simple and inexpensive pathway for the analysis of the genome as well as the production of unique fungal genotypes (Wenderoth et al. 2017; Wang et al. 2018). To overcome this barrier, scientists are now modifying CRISPR-Cas9 methods to alter the genomes of non-model fungi. Recently, CRISPR-based methods have been created for a number of filamentous fungi, including *Trichoderma* (Liu et al. 2015), *Penicillium* (Pohl et al. 2016), and *Neurospora* (Matsu-Ura et al. 2015). Other findings revealed that, editing of the fungal genome of *Pestalotiopsis fici* by the transformation of polyethylene glycol (PEG)-mediated protoplast using the CRISPR-Cas9 method resulted in the overproduction of SMs by the fungus (Xu et al. 2021; Wang et al. 2023). Another report has demonstrated that the gene silencing is responsible for stress signal of a mitogen-activated protein kinase (MAPK) which is done by using dual-gRNAs in the fungal endophyte *Phomopsis liquidambaris*. Various SMs belonging to the class of flavonoids, including quercetin, naringenin, and kaempferol, were biosynthesised as a result of this process, protecting the mutant cell against possible stressors (Huang et al. 2020). To summarise, endophytic fungi are promising candidates for producing novel SMs, so it is crucial that continuous research must be carried to assess the most recent discoveries to guide upcoming research in this field effectively. Functional identification and validation of biosynthetic gene clusters of physiologically functional polypeptides, enzymes, and chemicals is necessary to develop endophytic fungal biotechnology. These genomic clusters can be further activated, controlled, and regulated to enhance the products of interest and their subsequent novel applications once they have been suitably annotated.

## 6. Chemical modulation of the epigenetic machinery: manipulating gene expression through small molecules

Chemical epigenetic modifiers are small, naturally occurring, or artificial molecules that target epigenetic enzymes and change the epigenomics of organisms (Poças-Fonseca et al. 2020; Xu et al. 2021). These modifiers enhance the production of SMs by activating the cryptic biosynthetic gene clusters, thereby modulating the biosynthetic pathway. Previous studies have demonstrated epigenetic inducers including DNMT and histone deacetylase HDAC inhibitors induced revamping of cryptic metabolite production in a fungus, *Nigrospora sphaerica* (Lopes et al. 2012). Another report has shown exposure of a volatile organic compound-producing fungal endophyte, *Hypoxylon* sp. in which the production of new volatile SMs was elicited by the epigenetic modulators. The epigenetic-modulating compound such as AZA and SAHA which modulated the epigenetics of fungus and resulted in the altered VOCs profiles and bioactivities (Ul-Hassan et al. 2012). Another finding revealed that when fungal endophyte *Fusarium oxysporum* grown in culture media supplemented with SAHA, it stimulated the biosynthesis of SMs along with two novel fusaric acid derivatives (i) 5-(but9-enyl)-6-oxo-1,6-dihydropyridine-2-carboxylic acid (ii) 5-butyl-6-oxo-1,6-dihydropyridine-2-carboxylic acid (Chen et al. 2013). A previous finding has shown in their reports that the nutrient medium of fungal endophyte *Graphiopsis chlorocephala* supplemented with a small epigenetic modifier, NAD^+^ -dependent HDAC inhibitor, influenced the production of novel metabolites including 2-(2,6-dihydroxy-4-methylbenzoyl) 6-hydroxybenzoic acid, benzophenones and cephalanones A-F (Asai et al. 2013). The proteasome inhibitor bortezomib causes the production of a novel secondary metabolite by epigenetic modification of a filamentous fungus *Pleosporales* (VanderMolen et al. 2014). It has also been reported that a mangrove endophytic fungus *Phomopsis asparagi* DHS-48 resulted in the production of immunosuppressive agents as a result of epigenetic manipulation (Feng et al. 2022). The endophytic fungus, *Aspergillus niger* treated with 5-AZA (**1**) have been shown in a study to enhance the production of several volatile and non-volatile metabolites and also induces the production of novel SMs (Toghueo et al. 2016). Another report revealed that, biosynthesis of fumiquinazoline C (**12**) was enhanced due to the upregulation of biosynthetic gene clusters of an endophytic fungus *Aspergillus fumigatus* when the culture medium was supplemented with valproic acid (**7**) (Magotra et al. 2017). In one of the previous reports, the culture media of *Penicillium concavoradulozum* and *Aspergillus amstelodami* supplemented with chemical epigenetic modifiers such as quercetin and nicotinamide resulted in the enhanced production of secondary metabolite vinblastine (Gulyamova et al. 2019). A previous study reported that fungal endophyte *Xylaria psidii* mediated resveratrol (**15**) production was enhanced upon treatment with epigenetic elicitors, SBHA (**3**) (5 µm) and 10 µm 5-AZA (**1**) (Dwibedi et al. 2019).

### 6.1. Histone deacetylase inhibitors

There are at least four structurally distinct kinds of histone deacetylase inhibitors (HDACi): benzamides, aliphatic acids, cyclic peptides, and hydroxamates. In one of the study, filamentous fungus supplemented with HDACi [sodium butyrate (**4**), trichostatin A (**6**), and SAHA (**5**)]. These HDACi influence post-translational modifications in non-histone proteins and modify gene expression patterns (Kim and Bae 2011). Trichostatin A (TSA) (**6**) was first discovered in a bacterial strain, *Streptomyces hygroscopicus*, and is known to be involved in inhibiting HDACs as evidenced through several *in vitro* and *in vivo* studies. SAHA (**5**) has been derived from bishydroxamic acid and is an effective inhibitor of HDACs. The hydroxamic acid group in both TSA (**6**) and SAHA (**5**) binds to the Zn^++^ ion found in the active sites of Class I and II HDACs, inhibiting their function (Yoshida et al. 1990; Marks and Breslow 2007). Sodium butyrate is a naturally occurring substance that suppresses histone deacetylase activity even at millimolar doses. On the other hand, the effect of vorinostat, an HDAC inhibitor was also reported on the SM profile of two *Aspergillus* species (Aldholmi et al. 2020). Although the mechanism underlying this activity is undefined, existing literature suggest that sodium butyrate (**4**) act as HDACi in vicinity of hydrophobic pocket of the enzyme (Candido et al. 1978; Davie 2003).

### 6.2. DNA methyl transferase inhibitors

DNMT inhibitors often function through the mechanism of DNA-methylation-mediated silencing, resulting in a variety of modifications in developmental and other cellular processes, as well as novel phenotypic features. Different studies evaluated the reports for the use of DNMT inhibitors in a variety of filamentous fungi, including *Neurospora crassa* (Aramayo and Selker 2013; Aghcheh and Kubicek 2015). The Decitabine [5-aza-2′-deoxycytidine (**2**)] and 5-AZA (**1**) (DNMT inhibitor) are commonly used in investigations to clarify how DNA methylation affects the physiology of fungi. These synthetic medicines resemble cytidine because they have a nitrogen atom at 5^th^ position of the pyrimidine ring rather than carbon. As a result, the molecule gets integrated into the DNA and hinders the DNMT from properly transferring the methyl group. Subsequent DNA replication cycles as a result lead to passive demethylation. DNMTs are still attached to the DNA when DNMTi are present, and the proteasome pathway then breaks them down (Santi et al. 1984). The RNA molecule as well as the DNA to a lesser extent contain the ribonucleoside analogue 5-AZA (**1**). Only DNA contains the deoxyribose analogue decitabine (Gnyszka et al. 2013). In one of the previous reports, an endophytic fungi *Pestalotiopsis crassiuscula* grown in culture media supplemented with 5-azacytidine (**1**) have shown change in metabolic profiling along with the production of a novel compound coumarin validated through NMR spectra (Yang et al. 2014).

## 7. Eliciting epigenetic modifications: exploring physical, chemical, and biological methods

In addition to small chemical compounds, it has also been suggested that certain natural plant products and neutraceuticals, including turmeric (*Curcuma longa*), Brazilian nuts (*Bertholletia excelsa*), garlic (*Allium sativum*), grapes (*Vitis vinifera*), fava beans (*Vicia faba*), soybeans (*Glycine max*), green tea (*Camellia sinensis*), and cruciferous vegetables, can alter epigenetic modulation pathway (Vel Szic et al. 2010; Hardy and Tollefsbol 2011; Abdulla et al. 2013). By modifying histones and methylating DNA, these micronutrients are responsible for the activation of BGCs, similarly to pharmacologically active epigenetic elicitors (Meeran et al. 2010). In a previous report, Resveratrol (**15**), a polyphenol used as epigenetic modifier, has been found in grapes (Gehm et al. 1997; Papoutsis et al. 2010). Studies have shown that dietary substances from plants can alter the epigenome and enhance the production of SMs of fungal endophytes. Another team of researchers investigated the effects of turmeric extract and grape skin on the fungal endophyte *Colletotrichum gloeosporioides*, and the production of cryptic and antibiotic chemicals. The enhanced crude components were observed with 272.48% through addition of culture media with grape skin and with the supplementation of turmeric extract the enhancement was reported to be 174.32% when compared to respective control group (Sharma et al. 2017). These epigenetic regulators often stimulate other metabolic pathways while suppressing the activity of related enzymes in the biosynthesis process (Seyedsayamdost 2014).

## 8. Conclusion and future prospective

Epigenetic regulation of gene expression has attained fundamental importance in research field and has opened-up new horizons for the production of novel SMs (Sagita et al. 2021). Fungal endophytes are an important source of medicinally significant secondary metabolite belonging to large structural groups. The chemically diverse myco-derived metabolites have created an urge to explore fungal endophytes and decipher their potential in therapeutics compliant to scale-up commercially. The chemical epigenetic manipulation targeting chromatin structure and histones in fungal endophytes have been shown to contribute towards alteration of specialised metabolite profile to enhance chemo diversity. Epigenetic modification using small epigenetic modulators is an outstanding tool for screening novel fungal isolates, such as those from least explored habitats. By inducing chromatin modification, low molecular weight compounds like 5-AZA (**1**), SAHA (**5**), and valproic acid (**7**) play pivotal role in altering qualitative and quantitative production of cryptic specialised metabolites by endophytic fungi. These modifiers are involved in the modulation of transcriptional machinery thus regulates biosynthetic pathway for producing rare or novel compounds with higher yields. The method being inexpensive, facile and promising in terms of effectiveness can be used in laboratory conditions as well in biotech industry. Using CRISPR-Cas-based methods, genome editing of many fungal strains could be a promising approach for regulating an enhancing secondary metabolite production. However, few fungal strains have been subjected to CRISPR-based transcriptome modification and therefore extensive research is required in this field. The existing knowledge of fungal secondary metabolism is intended to improve by combining CRISPR-based RNA-targeting systems with the well-proven platform of CRISPR-based methods. A comprehensive study is still needed to effectively understand the regulatory pathways, genes or factors involved in the synthesis of fungal SMs along with the regulation of attenuation. The underlying mechanism of chemical modifiers remodelling epigenetics in fungal endophytes is crucial to understand so as to maintain the continuum of production of useful metabolites.
